# Sports Practice, Body Image Perception, and Factors Involved in Sporting Activity in Italian Schoolchildren

**DOI:** 10.3390/children10121850

**Published:** 2023-11-25

**Authors:** Luciana Zaccagni, Luca Rosa, Stefania Toselli, Emanuela Gualdi-Russo

**Affiliations:** 1 Department of Neuroscience and Rehabilitation, Faculty of Medicine, Pharmacy and Prevention, University of Ferrara, 44121 Ferrara, Italy; luciana.zaccagni@unife.it (L.Z.); luca.rosa@edu.unife.it (L.R.); 2 Center for Exercise Science and Sports, University of Ferrara, 44123 Ferrara, Italy; 3 Department for Life Quality Studies, University of Bologna, 47921 Rimini, Italy; stefania.toselli@unibo.it

**Keywords:** sports, children, expectancies and facilitators in sports, body image, BMI

## Abstract

Regular physical activity is generally deemed to positively affect health, but studies on children are scarce. Among the kinds of physical activity, sports practice is the most common and easiest to quantify and report by children. This cross-sectional study aimed to compare the two genders and evaluate the association between organized sports practice and body dissatisfaction in a sample of 214 Italian schoolchildren (55.6% males) aged 5 to 12. Body image perception and data on sports practice expectations and facilitators were collected in individual face-to-face interviews; weight and stature were directly measured. Girls tended to be sportier than boys (91.6% of girls vs. 86.3% of boys practiced sports), with an earlier start in sports (5.48 ± 1.47 vs. 5.72 ± 1.38 years) and a greater amount of weekly sports (3.41 ± 2.95 vs. 3.01 ± 2.11 h/week). In both genders, the ideal silhouette was more slender than the feel silhouette, and in girls more than in boys. According to the outcomes of multiple regression models, years of organized sports participation were a significant predictor of the weekly amount of sports in both genders, in addition to the feel weight status minus actual weight status inconsistency score, fun in sports, and parental support only in boys and teacher support only in girls. Children’s needs and interests and sports facilitators should be considered to promote an early active lifestyle.

## 1. Introduction

Physical activity exerts a beneficial effect on mental and physical health [1]. Undertaking it from childhood is essential and forms the basis of a healthy life [1]. The *UK Chief Medical Officers’ Physical Activity Guidelines* [2] suggest that it is critical for people from 5 to 18 years of age to minimize sedentary time and suggest moderate to vigorous intensity physical activity over the week for a minimum of 60 min a day. Children’s physical activities include active travel, structured sports, informal play, and domestic and leisure activities [3,4]. The benefits of active living include improving cardiovascular health, bone density, self-confidence, muscular strength and endurance, and body weight within a healthy range; they also enable the development of new social skills [2,5]. Many of the health benefits of a physically active lifestyle during the developmental stage carry over favorably into adulthood [6].

In addition to these general benefits that come from practicing physical activity, we can distinguish those that come from practicing unorganized or organized sports. While unorganized physical activities are self-organized, less structured, and freely controlled by other young people [7], organized sports are generally carried out within specific sports clubs or educational organizations; they are coordinated by adults and are characterized by a series of physical exercises, trapped individually or within a group, involving specific rules and formal practice [7,8,9]. The practice of organized sports is associated with positive developmental outcomes, including, for example, better academic achievement, physical performance, mental adaptation, and decreased rates of antisocial behavior, while self-organized physical activity enables the acquisition of social skills, including independence, cooperation, self-regulation, and problem-solving skills [7,10].

Moreover, following the literature, the practice of physical activity can be affected by numerous intrapersonal, interpersonal, environmental, cultural, socioeconomic, and political factors [11,12]. However, there is no doubt about the positive effect of an active lifestyle on strengthening the facilitators of such practice [12,13,14]. According to an international study involving adolescents from 146 countries around the world [6], adolescents from high-income countries were found to be more active than those from low-income countries. In high-income countries, such as the United States, the increased practice of physical activity in school-age individuals is likely related to improved school physical education, extensive media sports coverage, and a widespread presence of sports clubs offering the opportunity to practice many structured and organized sports [6]. A favorable family financial situation is associated with young people’s well-being and involvement in physical activity, providing more opportunities to participate in organized sports during leisure time [15,16]. Further barriers that decrease the support and opportunities available for children in sports are the low level of priority attributed to physical activity in the school setting and the behavior of parents [6,17]. In brief, costs, lack of time, lack of family support (e.g., encouragement), the distance to sports facilities, and peer or teacher disapproval may affect the child’s participation in sports activities [18,19]. In addition to these factors, children’s expectations of playing a sport have an important effect on their participation. In particular, sports dropout—frequent, especially in adolescence—depends mainly on the lack of enjoyment from playing sports and the perceived lack of competence in sports [5,20].

Physical education is regularly taught in all Italian elementary (1 h/week) and middle (2 h/week) schools to bring the child to knowledge of self, environment, and movement possibilities [21,22]. Outside the school setting, according to the Italian Istituto Superiore di Sanità [23], the continuative practice of sports characterizes the youngest groups of the Italian population and, in 2020, involved 58% of the 6–10-year-old group, 61% of 11–14-year-olds, and 50% of 15–17-year-olds. As part of the nationally and regionally relevant surveillance systems referable to the World Health Organization’s (WHO) European Childhood Obesity Surveillance Initiative—COSI, 3rd-grade children in 2467 Italian schools were surveyed nationwide in 2019, detecting unhealthy behaviors in 20.3% of children who had not been engaged in any physical activity the day preceding the survey. In addition, 20.4% of these children were found to be overweight and 9.4% obese, with males showing slightly higher obesity rates compared to females (obese boys: 9.9% vs. obese girls: 8.8%) [24]. This prevalence is worrisome since unhealthy weight status (defined by body mass index) has been found to affect both body image and physical activity practice between the ages of 6 and 11 years [25]. Overweight or obese children show problems with fundamental skills of movement and have high levels of dissatisfaction. The importance of the years of sports practice should also be emphasized, as early participation in sports practice is positively associated with self-esteem because it can promote physical competence and a favorable body image [1,26]. In pointing out that body image refers to a person’s perceptions and feelings about his or her body and that body image dissatisfaction can be determined when there is a discrepancy between perceived and ideal body image [27,28], regular physical activity has generally been found to be associated with a favorable body image [29] and perceived positive well-being [9,30,31]. During the growth process, changes in body image perception are controlled by gender and are particularly associated with the timing of puberty [32]. In particular, it is known that an unhealthy body image is associated with obesity and physical inactivity during adolescence [33]. Physical activity is effective for adolescents in enabling them to attain a positive self-concept and foster psychological well-being through improved body perceptions and body satisfaction [34]. This finding is consistent with the exercise and self-esteem model (EXSEM) [35] and its modification with BMI as a mediating variable [34], which explains how participation in these activities promotes overall self-esteem, enhances the sense of self-efficacy, and increases sports proficiency perception and bodily acceptance [36]. The association between body image and physical activity is complex and differs between the two genders, with greater levels of dissatisfaction in females than males [29,33,37]. In addition, while girls prefer a lean build, male adolescents prefer a muscular body image [38,39]. Although previous studies have shown that there is an association between physical activity practice and lower body dissatisfaction in adolescents [5,18,40], such studies on children and preadolescents are very scarce. Therefore, it is still to be clarified whether early sports practice is associated with lower body dissatisfaction and whether significant gender differences also exist during childhood. The paucity of studies on children in this research area, even though knowledge of body image development is crucial because many behaviors (eating and sports) are established early and have long-term consequences, is mainly caused by the belief that data on body image perception are sensitive and children constitute a fragile group [41]. However, according to recent research [41], it has been shown that this type of research is not harmful to the child and that data collected directly with the child’s participation have greater validity than data collected by interviewing parents or teachers. The need to develop research in this area on children also depends on the impossibility of extending the results obtained on adults and adolescents to children due to developmental differences both socially, biologically, and psychologically [42].

Thus, given the connection between physical activity practice, body image perception, and factors such as gender, this cross-sectional study intends to compare the two genders in a sample of children between childhood and preadolescent ages and evaluate the association between organized sports practice and body dissatisfaction, taking into account body weight status, years of sports practice, expectancies of rewards, and factors that facilitated the sporting activity.

## 2. Materials and Methods

### 2.1. Design, Procedure, and Study Participants

The present study was cross-sectional in design. This study received approval from the Bioethics Committee of the University of Bologna (approval n. 25027). The sample consisted of children attending elementary school and sixth grade in a town in northern Italy during the 2022–2023 school year. We carried out an a priori power analysis employing the G*Power statistical program (version 9.4; Heinrich-Heine-Universitat Dusseldorf, Dusseldorf, Germany) to calculate the sample size. The required smallest sample size for multiple regression analysis with nine independent variables was found to be 94 for 80% power at 90% confidence level and 0.10 significance level. Therefore, participants were 214 students (119 males and 95 females) aged 5 to 12 years old who were recruited from two elementary schools (first through fifth grades) and one secondary school (sixth grade). The selected schools had no access restrictions based on socioeconomic status, as is true for all Italian state schools, and were located in Monselice (Padua). The considered city has more than 17 thousand inhabitants and is located in the lower Po Valley in an area that is not densely populated. The largest number of enterprises in this area are active in agriculture, trade, construction, and industry, and the standard of living is medium–high [43,44]. All students of schools examined in the urban setting of Monselice were requested to be part of this study. Of the 218 pupils enrolled in the classes considered, we recruited only those who had received written consent with the signatures of parents or guardians and agreed to be involved. Four children (1.8%) were, therefore, excluded because they did not provide the prescribed consent. The study participation was entirely voluntary, and participants were guaranteed anonymity and confidentiality.

Participants were examined separately in a private area of the school to ensure privacy during anthropometric measurements and interviews.

### 2.2. Instruments and Variables

#### 2.2.1. Anthropometry

Anthropometric measures were collected by the same well-trained person in accordance with standardized procedures [45,46]: the stature was measured in the approximation of 0.1 cm by an anthropometer (Magnimeter, Raven Equipment Ltd., Dunmow, Essex, UK), and the weight was measured in the approximation of 0.5 kg by a mechanical scale (SECA, Basel, Switzerland). Both measurements were taken in the morning on children without shoes and in light clothing. Stature was measured on the child in a straightened position with the head in line with the plane of Frankfurt. Based on these two measurements, we calculated the body mass index (BMI): weight (kg)/stature^2^ (m^2^). The weight status of each child was assessed by BMI following Cole’s cut-off points as underweight, normal-weight, overweight, and obese [47,48].

#### 2.2.2. Demography, and Sports Practice

Individual face-to-face interviews were carried out by the same trained interviewer to collect basic biographical and demographic information: gender, birth date, any organized sport played, hours per week of sports practice, entry age in organized sports, expectations, and factors related to sports practice. In particular, physical activity practice was evaluated as organized sports because of the commitment and assiduity required [49]. This variable was measured in a dichotomous manner (yes/no) and as the number of hours per week. Years of organized sports participation were subsequently calculated from the difference between the date of surveys and the entry age of the participant, taking into account any discontinuity in such practice. Six simple and intuitive items assessed the expectancies of rewards and facilitators of playing sports in children according to the list shown in Table 1. The proposed items take into account previous literature [1,2]. For each item, there were two alternative responses: “I agree”, and “I disagree”.

The child’s perceived body image was evaluated by the interviewer with the use of Collins’ silhouette chart [50], which is the most widely used scale in children and has proven to be valid and reliable [51,52]. Children chose from seven figures of their gender, ranging from an emaciated to an obese figure, the one they perceived to be most similar to their body appearance (feel), and the one they would like to have (ideal). In particular, the figures were presented in a single chart in an ascending size sequence from left to right since it has been shown that the order of the figures does not affect selection [52]. Moreover, we proposed a modified chart with facial features removed from the silhouettes to avoid any influence on the choice [53]. By subtracting the ideal silhouette from the feel silhouette, we computed the feel minus ideal discrepancy score (FID) [54]. The discrepancy indicates the dissatisfaction level regarding the feel body image. Specifically, when the score is different from 0—a value indicating correspondence between the ideal and perceived figure—dissatisfaction denotes that the perceived body figure is more robust (score > 0) or leaner (score < 0) than the ideal.

Based on the mismatch of the perceived weight status, assessed by silhouettes chosen as feel, with the objective weight status, evaluated by BMI, we then computed the feel weight status minus the actual weight status inconsistency score (FAI) [3,55]. Specifically, we subtracted the code conventionally given to the child’s weight status (1: underweight; 2: normal weight; 3: overweight/obese) from the code of the feel silhouette: 1 when underweight (silhouettes 1 and 2); 2 when normal weight (silhouettes 3, 4, and 5); 3 when overweight/obese (silhouettes 6 and 7) [56,57]. When the score is other than 0—corresponding to no weight status perception inconsistency—the weight status may be overestimated (score > 0) or underestimated (score < 0).

There were no missing values in the anthropometric measurements or the sociodemographic, motivational, or body image perception data collected in the survey.

### 2.3. Statistical Analysis

We calculated descriptive statistics (means and standard deviations for quantitative variables and frequencies for qualitative ones) separately for males and females. The normal distribution of data was verified using the Kolmogorov–Smirnov test.

Gender comparisons for quantitative traits were carried out with an analysis of covariance (ANCOVA) controlling for age. For non-normally distributed variables, such as body image traits, we employed the U test of Mann–Whitney in the comparison between genders and the Wilcoxon matched pairs test in the comparison between the ideal and feel figures of the same subject within each gender. The comparisons for qualitative traits were performed using the chi-square (χ²) test.

Pearson’s or Spearman’s correlation coefficient (for normally or non-normally distributed variables, respectively) was calculated to determine the relationship between sports practice and the other quantitative variables. We carried out a backward multiple regression analysis adjusted for age to evaluate eventual predictors of sports practice (hours/week), selected as the dependent variable, separately by gender. The residuals were also analyzed (observed value minus predicted value for each case) to test the assumptions of multiple regression (independence, homoscedasticity, normal distribution of residuals). The independent variables selected were the following: years of organized sports participation, anthropometric and body perception indices (BMI, FID, FAI)—all entered into the model on a continuous scale—and expectations and facilitators for sports practice (agree = 0; disagree = 1)—included as binary variables. We assessed the multicollinearity of the data through variance inflation factors (VIFs), taking VIF values in the range of 0.10 to 10 as acceptable [58].

The effect size for each test was calculated: partial eta squared for ANCOVA, rank biserial correlation coefficient for U Mann–Whitney, Cramer’s V for the chi-square test, and Cohen’s f^2^ for multiple regression.

We performed the statistical analyses using “Statistica” for Windows, Version 11.0 (StatSoft Srl, Tulsa, OK, USA). We regarded values of *p* < 0.10 as statistically significant.

## 3. Results

Considering weight status (Table 1), the category of normal weight (NW) was prevalent in both genders (67.2% in males and 53.7% in females), but almost one-third of the sample was overweight (OW; 27.7% in males and 33.7% in females) (and in particular, a tenth was obese, O, 9.2% in males and 10.5% in females). The girls had a different distribution than the boys, as the underweight (UW) and OW/O classes were more numerous at the expense of the NW.

For what concerns body image perception, boys chose on average significantly more robust feel figures and ideal figures than girls. In both genders, the ideal figure was significantly more slender than the feel figure (*p* = 0.0052 in boys and *p* < 0.0001 in girls), so FID mean values were positive in both genders, indicating the desire to be slimmer; however, this was greater, though not significantly, in girls than in boys. Both genders tended to underestimate weight status, as the negative mean values of the FAI highlight.

**Table 1:** Characteristics of the overall sample and comparison between genders.

Variables	Total Sample (N = 214)		Boys (N = 119)		Girls (N = 95)		*p*-Value	Effect Size
***Anthropometric and BI perception indices***								
BMI (kg/m^2^)	18.00 ± 3.45		18.10 ± 3.27		17.88 ± 3.69		0.5377 *	0.002
Feel figure	3.69 ± 1.33		3.93 ± 1.27		3.38 ± 1.34		**0.0013** ^§^	0.219
Ideal figure	3.24 ± 1.25		3.58 ± 1.28		2.82 ± 1.08		**<0.0001** ^§^	0.310
FID	0.44 ± 1.33		0.35 ± 1.48		0.56 ± 1.12		0.1493 ^§^	0.098
FAI	−0.34 ± 0.59		−0.31 ± 0.56		−0.38 ± 0.62		0.3360 ^§^	0.066
***Weight status***	N (%)		N (%)		N (%)			
UW	18 (8.4)		6 (5.0)		12 (12.6)		**0.0546** ^&^	0.165
NW	131 (61.2)		80 (67.2)		51 (53.7)			
OW/O	65 (30.4)		33 (27.7)		32 (33.7)			
***Sports practice***								
Yes (N(%))	188 (87.9)		101 (84.9)		87 (91.6)		0.1358 ^&^	0.102
Amount (hours/week)	3.19 ± 2.52		3.01 ± 2.11		3.41 ± 2.95		0.2637 *	0.006
Sports practice (years)	2.93 ± 2.29		2.79 ± 2.21		3.11 ± 2.37		0.2744 *	0.006
*Playing Sports: expectancies of rewards and facilitators*	Agree N (%)	Disagree N (%)	Agree N (%)	Disagree N (%)	Agree N (%)	Disagree N (%)		
Makes fun	208 (97.2)	6 (2.8)	116 (97.5)	3 (2.5)	92 (96.8)	3 (3.2)	0.7792 ^&^	0.005
Makes health better	213 (99.5)	1 (0.5)	118 (99.2)	1 (0.8)	95 (100.0)	0 (0.0)	0.8726 ^&^	0.021
Makes thin	194 (90.7)	20 (9.3)	104 (87.4)	15 (12.6)	90 (94.7)	5 (5.3)	**0.0668** ^&^	0.230
Makes stronger	209 (97.7)	5 (2.3)	117 (98.3)	2 (1.7)	92 (96.8)	3 (3.2)	0.4772 ^&^	0.049
Finds support from my parents	198 (92.5)	16 (7.5)	110 (92.4)	9 (7.6)	88 (92.6)	7 (7.4)	0.9571 ^&^	0.003
Finds support from my teachers	172 (72.4)	42 (27.6)	95 (69.7)	24 (20.2)	77 (75.8)	18 (18.9)	0.8232 ^&^	0.003

* test ANCOVA adjusted for age; ^§^ U Mann test; ^&^ Chi-squared; UW = underweight, NW = normal weight, OW/O = overweight/obese. Bold values of *p* indicate statistical significance.

There was a tendency for girls to be more sporty than boys (91.6% vs. 84.9%), to start earlier with sports practice (5.48 ± 1.47 years in girls and 5.72 ± 1.38 years in boys), and to play a greater amount of sports weekly (3.11 ± 2.37 vs. 2.79 ± 2.21 h/week). A higher percentage of girls played two sports at the same time (13.7% of girls vs. 9.2% of boys). Soccer and swimming were the most practiced sports among boys, while dance, swimming, rhythmic gymnastics, and volleyball were the most played among girls.

Among the expectancies and facilitating factors of sports practice, almost all of both genders (percentages over 96%) agreed on the statements “Playing sports makes fun”, “Playing sports makes health better”, and “Playing sports makes stronger”. The genders differed significantly in the statement “Playing sports makes thin”, and boys disagreed more than girls with a medium effect size. The highest percentage of disagreement (over 20% in males) was recorded in the statement “Playing sports finds support from my teachers”.

There was a positive correlation of the weekly amount of sports with sports practice (r = 0.642 in males and 0.549 in females), BMI (r = 0192 in males), FID (ρ = 0.179 in males), FAI (ρ = 0.184 in males and 0.173 in females), and, in females, a negative correlation of the weekly amount of sports with BMI (r = −0.183) and FID (ρ = −0.208). All the correlations were significant (*p* < 0.10). Table 2 displays the results of multiple linear regression analysis for the weekly amount of sports as the dependent variable for boys. In the multivariate model, agreeing with the statements “Playing sports makes fun” and “Playing sports finds support from my parents”, years of organized sports participation and FAI were significant predictors positively associated with the dependent variable. The model accounted for 44.01% of the variance of weekly sports amounts in boys, with a large effect size (Cohen’s f^2^ = 0.849). The Durban–Watson value was 2.028, indicating no first-order linear auto-correlation in our multiple regression data. The other principal assumptions of multiple regression were also satisfied for both homoschedasticity (Figure S1) and the normal distribution of residuals (Kolmogorov–Smirnov *d* = 0.0674, p > 0.20).

Table 3 displays the results of multiple linear regression analysis in girls. In the multivariate model, agreeing with the statement “Playing sports finds support from my teachers”, and years of organized sports participation were significant predictors positively associated with the dependent variable. This model accounted for 32.40% of the variance of weekly sports amounts in girls, with a large effect size (Cohen’s f^2^ = 0.511). The Durban–Watson value for the test of independence was 2.113. The residuals were normally distributed (Kolmogorov–Smirnov d = 0.0838, *p* > 0.20), and homoscedasticity was verified (Figure S1).

## 4. Discussion

This study compared the two genders in a sample of children between the ages of childhood and preadolescence, analyzing the weekly sports practice in relation to years of organized sports participation, body image perception, BMI, expectancies, and facilitators for sports practice in male and female children. Outcomes indicated that the most informative predictor of the weekly amount of sports practice by the age-adjusted multiple regression was early participation in organized sports in both genders. The present study also revealed the different impacts of the examined facilitators on the two genders.

In addition, the comparative analysis carried out in this study between children of both genders indicated a rather homogeneous pattern in motivational and anthropometric characteristics but a different distribution in weight status. In contrast, the perception of body image showed significant differences between genders, except for the FID and FAI indices. The different feel figure and, especially, the ideal one with female concerns related to weight emerged from our comparison between genders consistently with the literature [56,59]. Confirming this, the analysis of expectations resulting from sports practice shows that girls believe that practicing sports enables them to achieve a leaner body by a significantly higher percentage than boys. Therefore, for females, the idea that sports practice determines real changes in the physical self (weight, body shape) seems to be an important motivation for practicing it. This outcome is not unexpected, as the literature points to the greater dissatisfaction of the female gender, which places greater importance on weight status than the male gender, following the lean standards of beauty fostered by the mass media in the Western world [34,60,61]. However, in the gender comparison, the degree of dissatisfaction with feel body image (FID) did not reach statistical significance in this sample of children, despite being higher in females compared to males. Although body dissatisfaction oriented toward thinness has already been reported in 6-year-old children [38], a critical phase will be determined thereafter, during adolescence, when body dissatisfaction will be affected by the interaction of body ideals with changes in the shape and size of the body [33]. In addition, the influence of mass media and especially social media, particularly in use among adolescents, may further contribute to increased dissatisfaction [62,63]. From a practical point of view, it should be noted that the figural scales used in this study, with their intuitiveness and quick handling, have proven to be a simple and cost-effective tool for evaluating bodily dissatisfaction in children.

It is interesting to note that almost all participants recognized the beneficial effects that playing sports can have on health, indicating the effectiveness of public health messages supporting the practice of physical activity both for preventive purposes and as a treatment of certain diseases. In Italy, some regions, such as Emilia-Romagna, Veneto, Lombardy, and Sicily, have for some time prepared together with the Ministry of Health a plan of health promotion interventions aimed at the entire population, including children, to spread more active lifestyles [64]. While males and females agreed on the beneficial health effects of playing sports, the major disagreement between genders was evidenced in the positive effect exerted by school support in sports practice.

Although in both genders early participation in organized sports emerged as the main predictor variable of weekly sports amount, a few differences in the regression models obtained in the two genders should be highlighted. In this respect, predictors of the amount of weekly sports practice in girls include school support in playing sports, while in boys, they include personal enjoyment in playing sports, family support, and the body perception index (FAI). Concerning this last variable, our study is in line with those of other researchers who have shown significant associations between moderate and intense activities and body image in adolescents and children [65,66]. In our study, in particular, sports practice increases as inconsistency in body image perception (overestimation) increases; the concern about being overweight would lead to increased sports practice. Therefore, the new element that emerged from our study is the different pattern in the two sexes, where the major facilitators of sports practice turn out to be the school in girls and the family and enjoyment of sports in boys. The largest effect size was found in the male gender.

These elements deserve careful consideration for the purpose of promoting sports in childhood. In fact, to promote active lifestyles, it is crucial to understand what expectations children have from sports practice and what factors facilitate it. According to a recent review [12], intrapersonal facilitators include greater physical activity levels associated with enjoyment in sports practice, while interpersonal facilitators include family or school staff support. In this study, the support of the school, through teachers, was found to be a factor that significantly affects the children’s sports practice only in girls, and this predictor variable, along with participation in organized sports, explained a total variance equal to 32.4%. On the other hand, in boys, sports practice is favorably affected by family support and the fun it generates. These predictor variables, together with participation in organized sports, explained a total variance of 44%. In both models, the *p*-values obtained in the multiple regression analysis indicated that the R-square provided a good degree of explanation for the weekly amount of sports practice.

According to a clinical report by the American Academy of Pediatrics [1], several benefits arise from organized sports. These are reported, as well as the roles of parents and schools in sports practice, indicating how participation in organized sports makes an effective contribution to a child’s physical, emotional, social, and psychological health. According to this report, the child’s perception of parental support is significantly associated with involvement in organized sports. On the other hand, parental awareness of the physical capabilities of their child in connection with their kid’s development and interests is crucial in deciding when to start organized sports. Positive support from family and school with encouragement, as well as attention to enjoyment and improvement in sports rather than achieving victory, represent valuable influencing factors [1].

Confirming the literature, even with different predictive models for the two genders, our findings may be useful to practitioners who should encourage children to participate in sports because of the positive health benefits and as a source of enjoyment. It also turned out that the social context (particularly parents and teachers) can play an important role in these choices during childhood. Here we emphasize the crucial role played by the teacher, who should not only teach the necessary techniques and tactics in different sports in school but rather should accustom students to exercise while gradually improving their sports culture following the sport education model [67]. In this regard, Tendinha et al. [68] reported in their review that some studies related to intrinsic motivation in physical education and sports have indicated that this construct is positively associated with the predisposition to participate in future physical activities. Other motivations related to maintaining body weight within a healthy range did not emerge among the main facilitators in this study and should be recalled with caution because body-related motives may be associated with disturbed eating attitudes and behaviors, as detected in adolescents [69]. More generally, we highlight the importance of the process of disseminating recommendations for an active lifestyle that ensures a child’s physical well-being. In this regard, the dissemination of guidelines for the children’s movement should involve stakeholders and policymakers who can develop and implement strategies for best practices in our society.

This study achieved established aims but is not without weaknesses. In particular, a major weakness is this study’s cross-sectional design, which prevents the determination of cause-and-effect relationships. Nevertheless, the elements that emerged are of some interest: they are relevant to the promotion of healthy behaviors and may inspire further research in the field, particularly longitudinal studies on numerically larger samples. The specific geographical area from which the sample was drawn suggests that the participants in this study may not be representative of the entire Italian child population. Therefore, the results obtained should be interpreted with caution. Future nationwide studies conducted on a representative population sample could confirm our outcomes. Another limitation is that data on sports practice were self-reported by the children. Therefore, we do not exclude an overestimation of the hours of sports practice, according to Rullestad et al. [70]. This study also did not consider the type of sport practiced. In this respect, we recall the importance that weight can have in some specific sports (e.g., rhythmic gymnastics), with possible effects on body image dissatisfaction [71,72]. However, it should also be noted that this dissatisfaction particularly concerns the ideal figure for that sport more than the ideal figure in everyday life [3]. Moreover, these aspects gain particular importance in elite athletes during adolescence, whereas childhood generally represents a start-up phase for sports practice. In terms of strengths, the most relevant in our study included the anthropometric measurements taken directly by the same expert operator, avoiding possible underestimation of being overweight or obese [73], and the face-to-face operator–child interaction that aimed to put each child at ease by assessing him or her separately from other children during the administration of the body image assessment test.

## 5. Conclusions

In conclusion, our results pointed out that the girls presented a more problematic situation than boys concerning weight status combined with higher body dissatisfaction due to a strong desire to have a slimmer body. Weekly sports practice amounts are predicted by early sports participation in both genders and, differently in the two genders, by body image and some expectations and facilitators. In particular, school staff support (in girls) and family support (in boys) seem important facilitators for sports participation in children, while enjoyment (in boys) represents the expectation children have from playing sports.

For the purpose of maintaining an active lifestyle from childhood, it is important to take into account children’s needs and interests and properly consider the facilitating factors.

## Figures and Tables

**Table 2:** Predictors of the weekly amount of sport in boys: multiple regression results using the backward method.

Predictor	VIF	β	t	*p*-Value
Sports participation (years)	1.0486	0.6076	8.6148	<0.0001
FAI	1.0109	0.1453	2.098	0.0381
Playing sports finds support from my parents (agree)	1.2060	0.1537	2.0317	0.0445
Playing sports makes fun (agree)	1.1715	0.1589	2.1306	0.0353
				
R^2^		0.4591		
R^2^ adjusted		0.4401		
p		<0.0001		

β: standardized regression coefficient; VIF: variance inflation factor; FAI: feel weight status minus actual weight status inconsistency.

**Table 3:** Predictors of the weekly amount of sport in girls: multiple regression results using the backward method.

Predictor	VIF	β	t	*p*-Value
Sports participation (years)	1.0949	0.6082	6.8542	<0.0001
Playing sports finds support from my teachers (agree)	1.0949	0.2010	2.2647	0.0259
				
R^2^		0.3384		
R^2^ adjusted		0.3240		
p		<0.0001		

β: standardized regression coefficient; VIF: variance inflation factor.

## Data Availability

Data are available upon request due to ethical restrictions regarding participant privacy. Requests for the data may be sent to the corresponding author.

## Supplementary Materials

The following supporting information can be downloaded at: https://www.mdpi.com/article/10.3390/children10121850/s1, Figure S1: Scatterplots of predicted values against residuals for girls (above) and boys (below).
