# Supplementary figures and images for: Sports Practice, Body Image Perception, and Factors Involved in Sporting Activity in Italian Schoolchildren
Source: Children (Basel). 2023 Nov 25;10(12):1850. doi: 10.3390/children10121850 (PMC10742091; doi:10.3390/children10121850)

Fig. S1: Scatter plots of predicted values against residuals for males (above) and females (below).

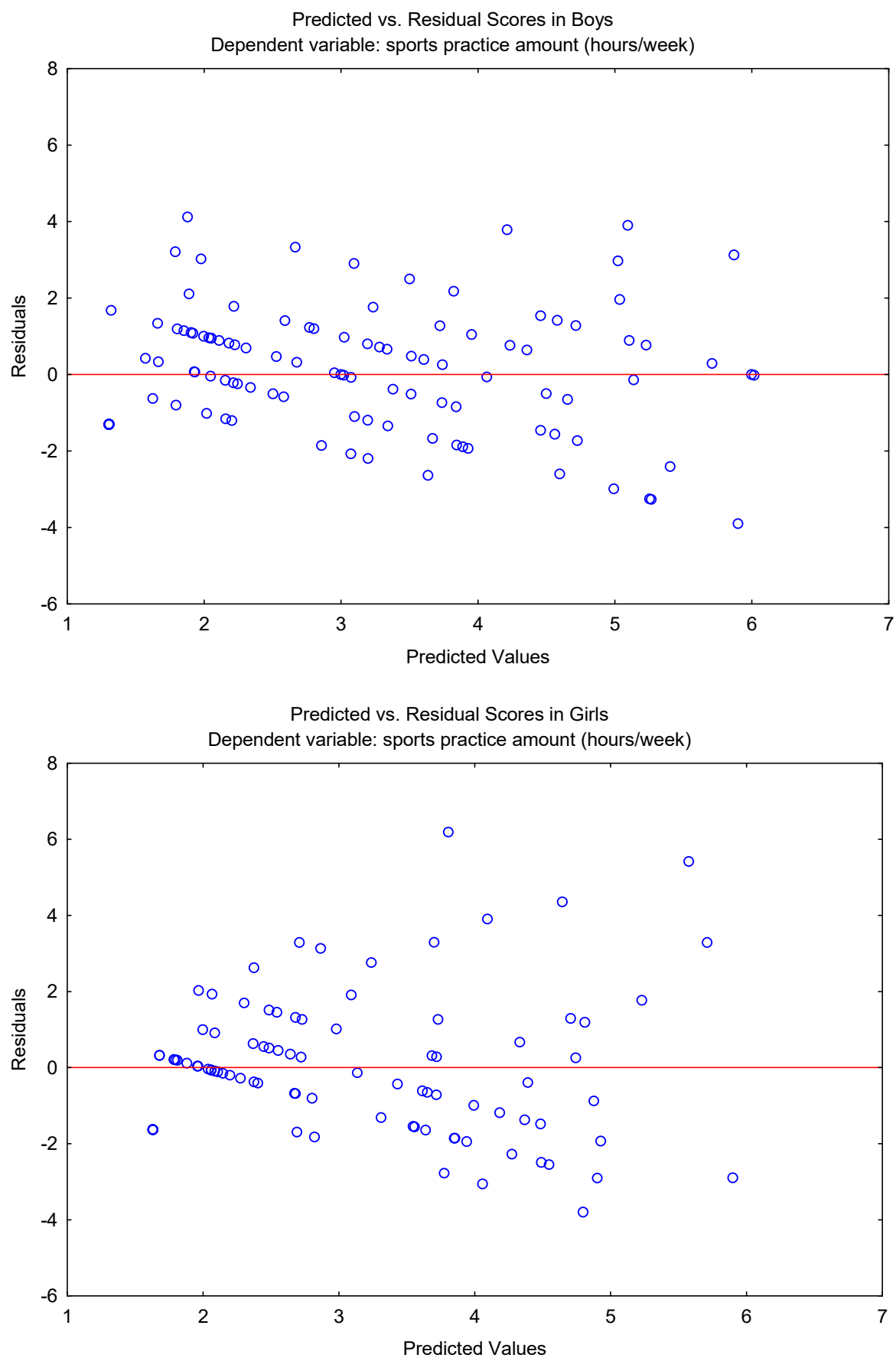

Supplement: Supplementary file 1 [file children-10-01850-s001.zip › children-2721458-supplementary.pdf]
